# Associations of the MIND Diet with Human Health Outcomes: A Scoping Review

**DOI:** 10.3390/nu17162687

**Published:** 2025-08-20

**Authors:** Katherine Hope Morgan, Michelle Lanphere Lee, Cristina S. Barroso, Joel G. Anderson, Shelley Lott, Danielle Reth, Chelsea Horn, Melanie Dixson

**Affiliations:** 1 College of Nursing, The University of Tennessee, 1200 Volunteer Blvd., Knoxville, TN 37996, USA; jande147@utk.edu (J.G.A.); slott4@utk.edu (S.L.); btl757@vols.utk.edu (D.R.); chorn10@vols.utk.edu (C.H.); 2 Department of Nutrition & Integrated Health Sciences, College of Health Sciences, East Tennessee State University, P.O. Box 70260, Johnson City, TN 37614, USA; leeml2@mail.etsu.edu; 3 College of Public Health, East Tennessee State University, P.O. Box 70623, Johnson City, TN 37614, USA; barrosoc@mail.etsu.edu; 4 Department of Health, Behavior, and Society, The University of Texas Health Science Center at San Antonio, 7703 Floyd Curl Drive, San Antonio, TX 78229, USA; 5 Health, Wellness, and Professional Programs, University Libraries, The University of Tennessee, 1015 Volunteer Boulevard, Knoxville, TN 37996, USA; melanie-dixson@utk.edu

**Keywords:** Mediterranean-DASH intervention for neurodegenerative delay, MIND diet, health outcomes, nutrition, risk reduction

## Abstract

The MIND diet was designed as an intervention to delay neurodegeneration and has been explored by systematic reviews for associations with cognition and, more recently, cardiometabolic disease. Comparatively less is known about how the MIND diet is associated with other health outcomes (e.g., all-cause mortality, anxiety, insomnia). This scoping review included studies exploring associations between the MIND diet and health outcomes other than cognition and cardiometabolic disease. Online databases were used to identify 4090 studies published between January 2015 and April 2024, from which 47 publications were included for review. Associations between the MIND diet and health outcomes were assessed as either favorable, unfavorable, or having no statistically significant association. Overall, 47 studies were included in this scoping review, 46 were observational, and several were conducted in large, established cohort studies. Across the 47 studies, 18 different topics were explored. Higher adherence to the MIND diet was mostly associated with favorable health outcomes (65%), while roughly one-third (33%) of studies found no statistically significant associations. One study, in Italy, found that increased adherence to the MIND diet was associated with increased exposure to cadmium, a heavy metal. In populations that may benefit from the MIND diet, we recommend additional observational and exploratory studies to identify health associations. Studies exploring educational interventions would help to identify facilitators and barriers to adopting the MIND diet. This scoping review provides some evidence that higher adherence to the MIND diet is associated with risk reduction for many diseases. Further research on environmental exposures (e.g., cadmium) and other deleterious substances absorbed by food crops will be crucial as we strive to enhance health and food security through plant-rich dietary patterns.

## 1. Introduction

Morris and colleagues designed the MIND diet (or the Mediterranean-DASH intervention for neurodegenerative delay diet) in 2015 [1,2] to delay the onset of Alzheimer’s disease and related dementias. The MIND diet is a hybrid between the Mediterranean and the Dietary Approaches to Stop Hypertension (DASH) diets. The MIND diet optimizes the intake of nutrients such as flavonoids, antioxidants, vitamins, and minerals that protect against inflammation and oxidation, which are key forces in neurodegeneration. Numerous studies have been conducted to test associations between the MIND dietary pattern and cognitive outcomes, and several research groups have conducted systematic reviews [3,4,5,6]. Likewise, the MIND diet has been systematically reviewed for associations with cardiovascular disease [7]. Overall, these reviews present evidence that the MIND diet is protective against dementia and beneficial to some domains of cognition and memory [3,4,5,6,8] and healthier cardiometabolic outcomes [7]. Comparatively less is known about how the MIND diet may influence other aspects of human health.

The MIND diet [1,2] consists of recommendations for quantities and types of foods to be eaten during a week (e.g., eating six servings of leafy greens) (see Table 1). Eating according to this dietary pattern provides ample plant materials (e.g., leafy greens), whole grains and starches (e.g., oats, rice, barley, sweet potatoes), beans and legumes (e.g., black beans, lentils), nuts (e.g., walnuts, cashews), berries (e.g., blueberries), seafood/fish (e.g., shrimp, fish), lean meats (e.g., chicken, turkey, venison), and plant oils (e.g., olive oil, canola oil). Morris [9] recommends limiting the number of red meats, saturated fats (e.g., whole dairy, lard, fried foods), and sweets consumed in a week, allowing for a few servings of these foods. The original MIND diet included “no more than” one daily serving of wine for women and two servings for men (i.e., 5 ounces of wine) [9].

Morris [9] wrote that one should fill their plate with “antioxidants, B vitamins, and healthy fats” (p. 35) to combat oxidative damage from free radicals or reactive species. Oxygen, nitrogen, and sulfur form reactive species and non-radical oxidants [10], which cause oxidative damage to DNA, lipids, and proteins [11]. Reactive species form naturally in plants and animals as the result of processes such as digestion, breathing, growing, and fighting infections [9]. Oxidative damage is associated with diseases including cancer, diabetes, neurodegeneration, and chronic inflammatory diseases [10]. However, the human body also produces antioxidants that offer protection against free radicals through a variety of mechanisms such as delaying or obstructing oxidative reactions, even acting as stabilizing agents by chelating metals [10]. Antioxidants include both antioxidant enzymes and nutrients [9]. Antioxidant nutrients, primarily vitamin E, carotenoids, flavonoids, other polyphenols, and vitamin C, are obtained through the foods that we eat [9]. The MIND diet was designed as a dietary pattern providing a variety of healthy foods, high in antioxidants, which support the body in its daily work of protecting against oxidative stress [1,2,9].

The bioavailability of dietary components depends in part upon the interplay between the foods we eat, commensal gut microbiota, and byproducts (metabolites) made by gut microbiota [12]. Changes in dietary patterns can quickly alter the community structure of the trillions of microbes inhabiting the human gut (bacteria, viruses, fungi, protozoans, and ancient Archaea) [13]. In a plant rich diet, the ample presence of flavonoids in the diet alters microbial community structure such that flavonoids are considered by some to be a prebiotic [14]. Recently, the microbially derived metabolite (produced by gut microorganisms), imidazole propionate (ImP), has been associated with subclinical atherosclerosis in mice and in humans, as well as multiple deleterious cardiometabolic measures such as dyslipidemia, visceral fat, and lower high-density lipoprotein (HDL) cholesterol, making ImP a new target for therapeutic interventions [15]. Interestingly, ImP was inversely associated with the Mediterranean dietary pattern, a largely plant-based diet and the foundation of the MIND diet [15]. Thus, dietary patterns, microbial community structure, and microbial byproducts may play important roles in disease prevention or progression.

Many noncommunicable diseases can be prevented or improved through lifestyle changes, such as adopting a healthier diet and exercising regularly [16,17]. Adherence to the MIND diet, especially in combination with physical activity, has been associated with cognitive benefits [18]. Plant-based diets are associated with improved cardiovascular health and all-cause mortality [19]. Understanding the extent to which the MIND diet may be beneficial would strengthen the knowledge base for making evidence-based recommendations about its use and adaptability to patient populations. For example, if a neuroprotective dietary pattern like the MIND diet can protect against dementia [1,2], then it is reasonable to suspect that it may be associated with benefits for other neurodegenerative diseases (e.g., glaucoma, multiple sclerosis, peripheral neuropathies, and Parkinson’s disease). The MIND diet promotes eating plant-based foods, including high fiber content, as well as healthy unsaturated fats, and lean meats [1,2]. Thus, the MIND diet could be beneficial for the prevention or treatment of many noncommunicable diseases such as hyperglycemia and obesity. However, it is unclear what kind of information is available in the literature about the MIND diet beyond the topics of cognition, dementia, and cardiovascular health.

The purpose of a scoping review is to map the studies published in an existing or new line of inquiry, to determine whether a systematic review is needed, and to identify gaps in knowledge [20]. In this scoping review, our aim was to identify peer-reviewed articles exploring adherence to the MIND dietary pattern and associations with health-related outcomes. Our primary research question was, in studies of humans in which adherence to the MIND diet was assessed, what were the health-related treatment outcomes, and did higher adherence to the MIND dietary pattern represent an improvement or a more favorable outcome than lower levels of adherence? Because the MIND dietary pattern recommends foods rich in antioxidants, we hypothesized that higher adherence to the MIND diet would be associated with improved health outcomes.

## 2. Materials and Methods

### 2.1. Protocol Registration

Our protocol followed the Preferred Reporting Items for Systematic Reviews and Meta-Analyses Extension for Scoping Reviews (PRISMA-ScR) [20]. The scoping review protocol was registered prospectively, prior to data abstraction, with Open Science Forum (Copyright © 2011–2025) [see registration at https://osf.io/bsvcm/?view_only=30c9fb317c30497892cd671be24ec5de (accessed on 12 December 2024)].

### 2.2. Information Sources

Collaborating with university research librarians, we designed search terms to identify articles published in English between January 2015 (the MIND diet was published in 2015) and April 2024 in peer-reviewed journals in which the MIND diet was the independent variable and any health outcome was the dependent variable (see Table 2). The following databases were queried: PubMed (including Medline; via the National Library of Medicine), Scopus (Elsevier), Web of Science (Clarivate Analytics), CINAHL (EBSCOhost), APA PsycINFO (ProQuest), and Google Scholar.

### 2.3. Selection of Sources of Evidence

To be included, a study must have been quantitative; published in English in a peer-reviewed journal; and conducted on humans. It was required to evaluate associations between the MIND diet score as the independent variable (e.g., where 0 is the lowest possible measure of adherence and 13 to 15 is the highest, depending on whether wine and olive oil were counted), and dependent variables included measurable health outcomes (e.g., longevity, mood, biometrics, muscle strength). We followed the Population Intervention Comparator Outcome (PICO) framework.

Excluded from this review were studies employing a modified Mediterranean-DASH diet, which was altered for other populations (e.g., the Korean-MIND diet); studies in which the dependent variables were the subject of previous systematic reviews related to cognition or cardiometabolic outcomes (unless other dependent variables such as mood were also measured); and studies where no health outcomes were measured, which have been recently reviewed [3,4,5,6,7]. Conference proceedings, dissertations, editorials, protocols, blogs, and reviews were excluded. Studies exploring only the Mediterranean diet or the DASH diet [21] (not the MIND diet, which is a hybridization of the two) were excluded.

### 2.4. Data Charting Process

References retrieved were downloaded from each database into EndNote™ version 21 (Clarivate), and then uploaded to Covidence^©^ (Veritas Health Innovation, Melbourne, Australia, Available at www.covidence.org), an online systematic review software in which research teams can conduct systematic and scoping reviews. While Covidence^©^ automatically removes most duplicates, some duplicates (e.g., a conference abstract and a manuscript of the same study) were removed manually. Within Covidence^©^, DR and CH independently conducted the initial review of titles and abstracts with KM serving as tiebreaker. The full-text review and data abstraction were conducted by CB, ML, SL, JA, and KM. Each text was reviewed independently by two reviewers, and in cases of a tie, a third reviewer. Questions were discussed through emails and teleconferencing until the full group reached consensus.

Data abstracted included the following for each selected article: country in which the study was conducted; participants’ demographic characteristics (patient population, disease, condition, age, gender); study design; who implemented the MIND diet or how the intervention was implemented (if any); what the intervention was and the time interval or duration of education about the MIND diet; how the MIND diet was scored and whether wine was assessed and scored; what methods of assessment and correlational analysis were used; and the covariates.

Critical appraisal is not recommended for a scoping review [20] and was not conducted. Abstracted data were downloaded to a Microsoft Excel for Microsoft 365 MSO (Version 2504) spreadsheet and organized into categories based on health outcome topics for evidence synthesis. For each health outcome, it was noted whether an association with the MIND diet was favorable, unfavorable, or no statistically significant association was found.

### 2.5. Synthesis of Results

All researchers conducted the evidence synthesis. Studies were grouped based upon related topics (e.g., dependent variables related to Parkinson’s disease). The evidence syntheses are presented in narrative format for each topic explored by two or more studies, including the types of settings, populations, and study designs, along with the measures used and broad findings.

## 3. Results

### 3.1. Identification and Selection

**Figure 1:**
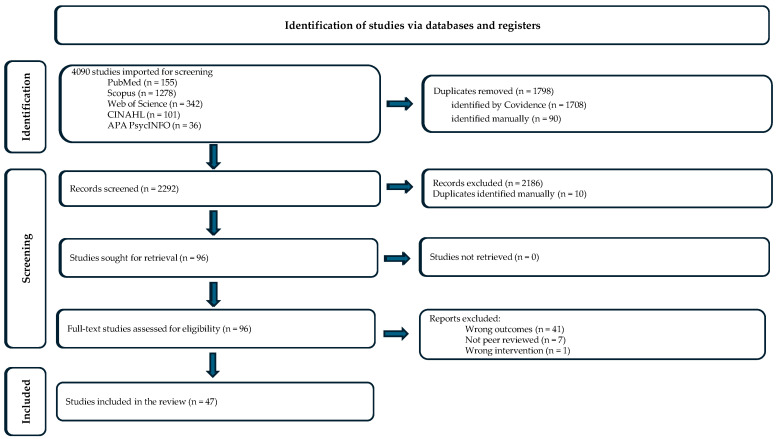
PRISMA diagram depicting selection of sources included in the scoping review.

The identification and selection process are depicted in a PRISMA diagram in Figure 1. The original search identified 4090 references. After removing duplicates and applying exclusion criteria, 96 full texts were assessed for eligibility and 47 studies met the inclusion criteria.

### 3.2. Study Characteristics

**Figure 2:**
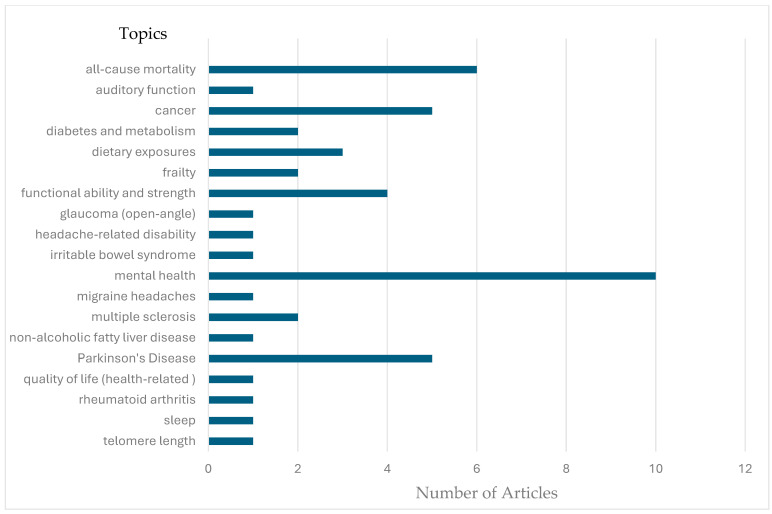
Number of articles by topic that were identified by this scoping review.

Table S1 summarizes the characteristics of the 47 studies. Studies were conducted in 10 different countries including Iran (*n* = 17), the U.S. (*n* = 10), China (*n* = 6), the U.K. (*n* = 5), Italy (*n* = 4), Spain (*n* = 2), Australia (*n* = 1), Canada (*n* = 1), and the Netherlands (*n* = 1) between 2018 and 2024. All but one [22] of the studies were observational. None of the studies included dietary education regarding the MIND dietary pattern, while one [22], using data collected by an ongoing cohort trial, provided an educational dietary intervention for the Mediterranean diet, but not the MIND diet specifically. Study samples ranged from 137 to 162,999 participants (median = 845, mean = 7824) and addressed 18 different health topics (see Figure 2).

Study designs included cross-sectional (49%), cohort (36%), and case–controls (15%). Longitudinal cohort studies ranged in the duration from 1 to 25 years. Some analyzed data from established cohort studies including the following: Australian Diabetes Obesity and Lifestyle study (AusDiab) [23], Baltimore Longitudinal Study of Aging (BLSA) [24], Framingham Offspring Cohort [25], National Health and Nutrition Examination Survey (NHANES) [26], Prevention with Mediterranean Diet (PREDIMED)-plus-Cognition [22], Reserve against Disability in Early Multiple Sclerosis (RADIEMS) [27], Rotterdam Study [28], Rush Memory and Aging Project (MAP) [29], Scottish Mental Survey [30], the Seguimiento Universidad de Navarra (University of Navarra follow-up, SUN) project [31], and UK Biobank [32].

To inventory food intake, most researchers relied upon validated food frequency questionnaires (FFQs) completed by study participants. These FFQs quantify eating patterns over a specified period, typically for the previous 12 months. Some researchers utilized FFQs that were validated specifically for cultural relevancy, such as the Chinese FFQ used by Zhang, He [33]. Four research groups completed FFQs by interviewing participants [22,34,35,36].

All but one research group calculated a MIND diet score (MDS) to measure adherence by following the method published by Morris et al. [1], where the minimum (no adherence) was 0 and the maximum score (highest adherence) was 15 (based on 15 food groups). Within each of the 15 food groups, points (0, 0.5, or 1) were awarded based on meeting the recommended servings/frequency of foods consumed for each food group per week. Twenty-six research groups (55%) used the original 15-point scale. In some cases, the FFQ did not adequately inventory dietary intake for some food groups (e.g., wine, olive oil, butter, or fried foods). In other cases, there were cultural constraints against some categories, such as wine. When one food category was not inventoried (e.g., wine), a 14-point MDS was employed (*n* = 13, 28%). Similarly, when two food groups were not included, a 13-point scale (*n* = 5, 11%) was utilized. Two studies used a 9-point scale. One study used a 14-point scale, but expanded the points awarded per food group to a range of one to five (where 1 is lowest and 5 is highest adherence), instead of 0, 0.5, or 1 per food group, for a possible range of 14–70 points.

For statistical analyses, research groups treated the MDS as an ordinal or a nominal value by creating tertiles of low, medium, and high adherence (e.g., where 0–5 is low, 6–10 is medium, and 11–15 is high on a 15-point scale). Others binned data into quartiles or quintiles. Research groups employed a variety of methods to test for statistically significant associations and differences between the MIND diet and health outcomes of interest (see Table S1).

The majority of health outcomes associated with higher adherence to the MIND dietary pattern were favorable (65%), while one-third (33%) reported no association or only a weakly favorable association. Only one of the studies included in this review identified an unfavorable outcome associated with higher levels of adherence to the MIND dietary pattern (i.e., increased urinary cadmium levels) [37]. Table 3 summarizes the associations between MDS and health outcomes reported in the 47 studies included in this review. Among studies (*n* = 20) where it was possible to identify the lowest MDS necessary to achieve a statistically favorable outcome, the average low score was 8.7 (median = 9.3; range = 6.0 to 10.6).

### 3.3. Evidence Synthesis of Similar Studies

This section synthesizes the available evidence for which more than one study focused on a similar health outcome (e.g., depressive symptoms) and an MDS was tested for associations with the health outcome(s) of interest.

#### 3.3.1. All-Cause Mortality

Six of the reviewed articles [25,26,30,38,39,40] examined associations between the MIND diet and mortality. Four research groups [25,26,38] found a statistically significant relationship with higher adherence to the MIND diet and a decrease in deaths or risk of mortality. However, Zupo, Sardone [39] found only a borderline association. Chan, Yu [40] found no association between MIND diet adherence and risk of mortality.

Three of the studies, examining the MIND diet and mortality, were based in the U.S. using nationally representative samples. Song, Chang [26] included 6887 patients from NHANES. The sample consisted of an average age of 47.13 ± 0.45, approximately equal numbers of males and females, more than 50% non-Hispanic Whites, approximately equal proportions of Blacks (19.36%) and Mexican Americans (18.47%), and approximately 50% nonsmokers, with 43.33% reporting hypertension, and 71.93% reporting hyperlipidemia. In this prospective cohort study, patients were followed for 10 years to record all cause and cerebrovascular (CV) deaths in patients with or without diabetes. Patients with a comorbidity of diabetes and high MDS (i.e., >8) experienced the lowest risk of all-cause mortality (HR = 0.75, 95% CI = 0.59–0.96, *p* = 0.021) and CV mortality (HR = 0.50, 95% CI = 0.29–0.87, *p* = 0.014) compared with patients with diabetes and lower adherence to the MIND diet. Patients without diabetes and high MDS also experienced a decrease in all-cause mortality (HR = 0.83, 95% CI = 0.70–0.99, *p* < 0.0001) but no statistically significant difference was found among patients without diabetes in terms of high MDS and CV mortality. When adjusting for all covariates, non-diabetic and diabetic patients with high MDS experienced the lowest risk of all-cause mortality.

In a similar analysis, Song, Chang [38] examined associations between hypertension, mortality, and MDS in a subset of 2984 participants from NHANES. Those with hypertension and high MDS (≥8.5) experienced a decrease in risk of all-cause mortality (HR = 0.69, 95% CI = 0.58–0.81, *p* < 0.0001) and CV mortality (HR = 0.62, 95% CI = 0.46 –0.85, *p* for trend = 0.0001) in comparison with participants with hypertension and low MDS (<7.5). For every point increase in MDS, participants had a 10% decrease in all-cause death (HR = 0.90, 95% CI = 0.86–0.95, *p* trend <0.001) and a 13% decrease in risk of CV death (HR = 0.87, 95% CI = 0.79–0.96, *p* trend = 0.001). Those without hypertension displayed an inverse relationship between MDS and all-cause death, but the relationship was not statistically significant. Thus, the comorbidity of hypertension and diabetes and a low MDS was associated with a significant increase in all-cause deaths in both studies.

Three research groups [25,30,39] also demonstrated a reduction in the risk of death among participants with high MDS. Thomas, Ryan [25] examined 1644 participants, offspring of the Framingham Heart Study (an U.S. based cohort of participants studied since 1948) in a prospective cohort study for 20 years. The average age of the population was 69.6 ± 6.9 years, approximately equal female and Male, and 53.3% smokers. Fewer than 20% of participants had cardiovascular disease (18.6%) and/or diabetes (16.8%). MIND diet scores were grouped into tertiles, low MDS (<6.5), moderate adherence (6.5–8) and high MDS (>8). Like the previous U.S. population-based studies, a lower risk of mortality correlated with higher MIND diet adherence (each 1 SD increase in MDS was linked to 47.3 [−74.8; −20] fewer deaths per 10,000 years of follow up). Thomas, Ryan [25] study demonstrated a correlation of 57% of the MIND diet score on mortality risk was also linked to the pace of age as measured by DunedinPACE (β_NDE_ + 20.1 [−47.2; 6.9] and β_NIE_ = −27.2 [−36.7; −18.8]). Thus, a slower pace of aging also impacts the risk of mortality among patients with higher adherence to the MIND diet.

In terms of studies conducted outside the U.S., Corley [30] examined adherence to the MIND diet over 12 years using the longitudinal Lothian Birth Cohort in Edinburgh, Scotland. The average age of the sample was 69.6 years with equal proportions of males and females and an average BMI of 27.1. The majority of the sample were smokers (57.1%) and moderately active (65.6%). Roughly one in five reported a history of CVD (21.4%), 37% hypertension, 5.2% diabetes, and 3.0% stroke. Those with the highest tertile MDS had a reduced risk of death by 37% (HR = 0.63, 95% CI: 0.41–0.96, *p* = 0.03). An inverse relationship was observed between adherence to the MIND diet and all-cause mortality. After adjusting for covariates such as age, BMI, hypertension, CVD, diabetes, and stroke, only the MIND diet had a significant association with mortality (HR = 0.88, 95% CI = 0.79–0.97, *p* = 0.01); the risk of death decreased by 12% per point increase in MDS.

Two other studies conducted outside the U.S. found borderline or no association between the MIND diet and mortality. Zupo et al. [39] examined 2472 participants in southern Italy in a retrospective cohort study. This sample consisted of 42.9% females and 31.88% smokers, with an average BMI of 27.48 ± 4.55. A small proportion (6.23%) reported comorbidities (diabetes, hypertension, peptic ulcer, cholangiolithiasis, myocardial infarction, hepatic cirrhosis, or other liver diseases). MDS demonstrated only a borderline inverse association with mortality (HR = 0.95, 95% CI = 0.92–1.00).

Chan, Yu [40] examined the MIND diet and mortality among 2802 participants in a prospective cohort study in Hong Kong and found no association between adherence to the MIND diet and mortality. However, more women in the low adherence group compared with the high MIND diet adherence reported higher incidence of diabetes (*n* = 79, 17.0%; *n* = 44, 9.4%, respectively) and cardiovascular events (*n* = 91, 19.6%; *n* = 73, 15.7%, respectively).

#### 3.3.2. Cancer/Oncology

##### Breast Cancer

Three case–control studies explored associations between adherence to the MIND diet and the risk of developing breast cancer (BCA) [36,42,43]. Two studies found significant associations [36,42], while one did not [43]. All were conducted in Iran—one in Isfahan [42] and two in Tehran [36,43]. Aghamohammadi et al. [42] and Mokhtari et al. [36] studied women ≥ 30 years of age who had recently been diagnosed with BCA (cases) and healthy women of the same age (controls). Aghamohammadi et al. enrolled 350 cases and 700 age-matched controls, and Mokhtari et al. enrolled 136 cases and 272 age-matched controls. Both studies found that higher adherence to the MIND diet lowered the odds of BCA significantly. After adjusting for possible confounders, women with the highest adherence to the MIND diet had 50% lower odds of BCA than those in the lowest tertile of MDS (OR = 0.50; 95% CI = 0.34–0.72 [42] or 45% lower risk (OR = 0.55, 95% CI = 0.32–0.96) [36]. Post-menopausal women with the highest MDS were less likely to have BCA than those in the bottom tertile, and there was an inverse association between high MDS and BCA among women of normal weight [42]. High MDS was also significantly associated with a lowered risk of BCA among women with a history of abortion [69]. Authors of both studies noted that case–control studies cannot prove causality and can be subject to selection and recall bias. By contrast, in a case–control study of 300 women (150 BCA cases and 150 age-matched controls having no relation with the cases), Sheikhhossein, Imani [43] found no significant association between high MDS and lower risk of BCA, even after controlling for potentially confounding variables (OR = 1.32, 95% CI = 0.31–5.64, *p*-trend = 0.633).

##### Gliomas

Gliomas are the most common type of malignant brain tumors found in adults [70]. Two research groups investigated associations between the MIND diet and the risk of glioma [33,44]. Both were case–control studies. One was conducted in Tehran, Iran [44] and the other in Beijing, China [33]. In Tehran, Soltani, Shayanfar [44] found that after controlling for age, energy intake, and gender, those with an MDS in the highest tertile had a 47% reduction in likelihood of having a glioma than those in the lowest tertile (OR = 0.53, 95% CI= 0.30–0.94). In their study of 1012 participants in Beijing, Zhang, He [33] found that four of the five diets tested reduced the risk of glioma, including the MIND diet (OR = 0.25; 95% CI= 0.14–0.44). The MIND diet also demonstrated a linear dose–response relationship between increasing adherence and reduction in risk of glioma. Furthermore, they discovered that certain diets, including the MIND diet, lowered the risk of various subtypes of glioma.

#### 3.3.3. Diabetes and Metabolism

Using different approaches and computational measures, two different research groups found strong associations between high MDS and lowered odds of having either a metabolically unhealthy phenotype [45] or type 2 diabetes [46].

Tirani, Poursalehi [45] examined the relationship between the MIND diet and metabolic health status (hypertension, hypertriglyceridemia, hyperglycemia/insulin resistance, and chronic inflammation) relative to serum concentrations of brain-derived neurotrophic factor (BDNF) (a neurotrophic factor that may also play a role in systemic energy metabolism [71]). This cross-sectional study with 527 adults (286 males, 241 females) aged 20–65 conducted in Isfahan, Iran, utilized a validated 168-item FFQ to collect participants’ dietary intake and to estimate their MDS (0–14, wine not included). Participants also were assessed for blood pressure, anthropometric measures (weight, height, BMI, waist circumference, body composition) and biochemical parameters. Tirani, Poursalehi [45] created a metabolically unhealthy phenotype based on hypertension, hypertriglyceridemia, hyperglycemia/insulin resistance, and chronic inflammation. Logistic regression evaluated the relationship between the MDS and the metabolically unhealthy phenotype. Tirani et al. [45] reported 58% lower risk of a metabolically unhealthy phenotype in participants with the highest MDS in their adjusted model (OR = 0.42, 95% CI = 0.20–0.90). This inverse relationship conferred even less risk for females and normal weight participants (OR = 0.19, 95% CI= 0.04–0.83), while for males there was no significant relationship between MDS and the metabolically unhealthy phenotype in the crude or adjusted models. Additionally, Tirani et al. found significant inverse associations between high MDS and hypertension (OR = 0.43, 95% CI = 0.19–0.94) and hypertriglyceridemia (OR = 0.21, 95% CI = 0.21–1.00). No significant association was observed between high MDS and serum concentrations of BDNF.

Tison, Shikany [46] compared dietary patterns (Mediterranean diet score, DASH diet score, MIND diet score, dietary inflammation score [DIS], and dietary inflammatory index [DII]) and risk of diabetes in a cohort of Black and White adults in the US using data from the REasons for Geographic and Racial Differences in Stroke (REGARDS) prospective cohort study. Demographic characteristics, cardiovascular risk profiles, blood pressure, fasting blood glucose, urine samples, electrocardiogram, and medication inventory of 30,239 Black and White adults, aged 45 years and older, were collected at baseline (from 2003 to 2007), and from 14,448 Black and White adults at a second in-home visit (from 2013 to 2016). Only data from participants with information about diabetes status at the second in-home visit were used (cross-sectional study design). Dietary assessment was conducted using the 107-item Block98 FFQ. Participants’ mean age was 63.2 ± 8.5 years, 56.2% were female, and 27.1% were Black. Most participants with diabetes were Black, male, had lower levels of income, had fewer years of education, were smokers, and had elevated waist circumference. Modified Poisson regression assessed the association between dietary measures (MDS) and risk of incident type 2 diabetes, with models adjusted for total energy intake, demographics, lifestyle factors, and waist circumference. Tison et al. [46] reported a statistically significant association between the MDS and incident type 2 diabetes (RR = 1.33, 95% CI = 1.07, 1.65; *p* = 0.02). A low MDS was strongly associated with incident diabetes. Based on their findings, the authors suggest that the MIND diet may be more effective in reducing the risk of incident type 2 diabetes compared with the Mediterranean and DASH diets.

#### 3.3.4. Dietary Exposures

Through foods ingested, humans are exposed to advantageous or deleterious environmental substances, based upon where and how a crop is grown (e.g., soil, water, fertilizer, insecticides). Three studies were conducted in Italy regarding uptake of sulfur [47], selenium [48], and cadmium [37] relative to MIND diet adherence.

##### Sulfur

Sulfur is an essential mineral required by humans daily. Passafiume, Rossetti [47] measured sulfur content in food samples and assessed its habitual intake in relation to adherence to the MIND diet among 719 adults in Northern Italy. Adherence to the MIND diet had a positive association with sulfur intake. This study provided an overview of sulfur content in foods composing the Italian diet (e.g., broccoli, kale), which are a significant component of the MIND diet.

##### Selenium

Selenium is a trace element found in many chemical forms and has nutritional and toxicological properties, some of which may play a role in the etiology of neurological disease. Urbano, Filippini [48] investigated the extent to which adherence to the MIND diet in a healthy non-smoking samples (*n* = 137) may be associated with selenium exposure, as ascertained by dietary, urine, and serum measures. Their results suggest that a greater adherence to the MIND diet is positively associated with lower circulating concentrations of selenium and of two potentially neurotoxic species of selenium, selenoprotein P and selenate. Researchers noted this may explain why adherence to the MIND dietary pattern may reduce cognitive decline.

##### Cadmium

Cadmium is a toxic heavy metal with detrimental effects on human health. In the U.S., the Food & Drug Administration regulates levels of cadmium in foods, especially those consumed by young children such as breads and cereals [72]. The main exposures to cadmium include diet (e.g., cereals, vegetables, and other plant-based foods), smoking, and occupational factors. Cadmium is present in the soil in greater quantities when phosphate fertilizers are applied, and in areas of heavy industry such as mining and smelting [72]. The relationship between adherence to the MIND diet and cadmium exposure was assessed for the first time and evaluated through urinary levels in an Italian cohort study of non-smoking, healthy blood donors [37]. Urbano [37] reported that higher adherence to the MIND diet was associated with higher cadmium exposure.

#### 3.3.5. Frailty

Frailty is a recognized clinical condition, defined by meeting three out of five measures of low function in the following criteria: energy, grip strength, walking speed, unintentional physical activity, and/or unintended weight loss [73]. Two studies assessed the MIND diet for associations with frailty [24,32]. Yao, Jia [32] analyzed data from the UK Biobank (UKB) for participants (mean age of 57.7 years) who had undergone two or more 24 h dietary assessments and had frailty data (*n* = 124,261) as measured by the frailty phenotype and the frailty index (FI_Frailty by Mitnitski, Mogilner [74]). The middle and upper tertiles of MDS (and those of the other healthy diets they assessed) had significantly fewer cases of frailty. For example, in their fully adjusted model, the highest tertile of MDS conferred 31% less risk of frailty compared with the lowest tertile, for frailty phenotype (OR = 0.47, CI = 0.63–0.76, *p* < 0.001) and 26% lowered odds of frailty on the frailty index (OR = 0.74, CI = 0.70–0.78), *p* < 0.001). This study also sought to determine whether metabolites mediate the association between diet and frailty. Participants who were missing data for plasma metabolites or creatinine were excluded from the mediation analysis (*n* = 97,991), leaving 26,270 participants whose metabolomic data were analyzed. Yao, Jia [32] found that metabolic signatures for each diet partially mediated the associations of dietary patterns with frailty. In a previous study, Tanaka, Talegawkar [24] analyzed data from a subset of participants (*n* = 806) aged ≥ 65 years, from the Baltimore Longitudinal Study of Aging (BLSA) cohort, which has been enrolling adults living in the community of the Washington, DC-Baltimore area in the U.S. Frailty was measured by 44 variables selected from the Frailty Index (described by Searle, Mitnitski [75]). Tanaka, Talegawkar [24] found that for the healthy dietary indices tested, MIND, Alternative Healthy Eating Index [AHEI], Mediterranean diet score, all were inversely associated with the Frailty Index (for MIND, β = −0.006 ± 0.002, *p* = 0.005). They also found that metabolic signatures for MIND and AHEI mediated the association between their respective dietary patterns and Frailty Index.

#### 3.3.6. Functional Ability

Four different research groups found positive correlations between adherence to the MIND diet and functional ability. In a study that followed participants for an average of 5.3 years, Agarwal, Wang [49] reported that participants in the second and third tertiles of MDS had a lower risk of developing disability affecting activities of daily living (ADL) compared with participants in the lowest tertile. They also discovered that the MIND diet was inversely associated with disability in instrumental ADLs (as measured by the Duke Instrumental ADL for independence with higher order tasks such as medication management) and mobility (as assessed by the Rosow-Breslau mobility index, which assesses the need for help with tasks such as walking up and down stairs and performing heavy housework). A cross-sectional study performed by Pasdar, Moradi [50] found that a higher MDS was associated with increased grip strength, and Talegawkar, Jin [51], another study that assessed data from a longitudinal aging study found similar results. Talegawkar, Jin [51] also reported that MDS was inversely associated with physical function impairment, and that participants in the highest tertile of the MDS had a 57% lower chance of functional impairment compared with the lowest tertile. In a cross-sectional study conducted in Hong Kong, Yeung, Sin [52] found that a higher MDS was associated with greater psychological function among participants.

#### 3.3.7. Mental Health

Ten different studies addressed aspects of mental health including anxiety [54,55,56,57], depression [29,31,54,55,56,57], impulsivity [22], mood [59], psychological stress [54,57], somatization [60], and stress [55,56]. Overall, the findings were equivocal, with some finding favorable associations between high MDS and the odds of a particular mental health outcome and others finding no association. Nine of the studies were conducted among Iranian cohorts, one in the U.S.; most were cross-sectional. Two studies focused on depression longitudinally [29,31]. Single studies were conducted regarding impulsivity [22] and mood [59], and neither found a statistically significant association with the MIND diet. A single study of somatization [60] conducted among 2818 adults in Isfahan, Iran, demonstrated a favorable association between high MDS and a reduced risk of psychosomatic and some somatic complaints.

##### Anxiety

Two research groups found significantly favorable associations, while three did not find a significant association between high MDS and lowered odds of anxiety. All five studies were conducted in Iran among different study populations. Torabynasab, Shahinfar [58] conducted a case–control study with 85 cases and 170 controls to explore associations between MDS with odds and severity of anxiety disorders. Higher MDS had an inverse association with severity of anxiety as measured by the Generalized Anxiety Disorder-7 (GAD-7) questionnaire (β = −3.63, *p* < 0.001) and a 97% reduction in odds of anxiety for those in the top category of MDS (OR = 0.03, 95% CI = 0.01–0.09, *p* < 0.001). Barkhordari, Namayandeh [54] conducted a cross-sectional study (*n* = 7165 participants) demonstrating a significantly lower odds of symptoms of anxiety [as measured by the Depression Anxiety Stress Scale-21 (DASS-21)] for individuals with the highest MDS (OR = 0.61, 95% CI = 0.41–0.91, *p*-trend = 0.01). Studying male health professionals (*n* = 400), Rostami, Parastouei [55] found no significant associations between MDS and odds of symptoms of anxiety, depression, or stress, as measured by the DASS-21. In a cross-sectional study of 282 women without underlying diseases or malignancies in Tehran, Iran, Seifollahi, Sardari [56] found no significant associations with symptoms of anxiety, depression [although depression was close to significant in the adjusted model (*p* = 0.068)], or stress, while they did find a significantly inverse association between highest MDS and psychological stress (OR = 0.87, 95% CI = 0.7–1.09, *p* = 0.23). Salari-Moghaddam, Keshteli [57] conducted a cross-sectional study with 3176 adults and used the Iranian version of the Hospital Anxiety and Depression Scale (HADS) to assess the severity of anxiety and depression. In their adjusted model, the highest MDS quartile was associated with a reduced odds of symptoms of depression (OR = 068, 95% CI = 0.53–0.89) and psychological distress (OR = 0.68, 95% CI = 0.52–0.89) compared with the lowest MDS quartile; however, they did not find a significant association between MIND diet and level of anxiety.

##### Depression

Results for high MDS and reduced odds of depression were equivocal, with half of the studies demonstrating a significant reduction in symptoms of depression [29,54,57] and half finding no association [31,55,56]. Working with 7165 participants in the Yazd Health and Yazd Nutrition study cohorts, Barkhordari, Namayandeh [54] found that the highest quartile of MDS was associated with significantly lower odds of depressive symptoms in the adjusted model (OR = 0.62, 95% CI = 0.40–0.96; *p*-trend = 0.02). In a longitudinal study of 709 participants over 6.53 years within the Rush Memory and Aging Project (MAP) in the U.S., Cherian, Wang [29] observed that the highest tertiles of MDS had lower rates of symptoms of depression compared with those in the lowest tertiles (β = −0.12, CI: −0.23, −0.0092). Seifollahi, Sardari [56] noted a trend demonstrating that the MIND diet is inversely associated with odds of depression (*p*-trend = 0.068). By contrast, Fresan, Bes-Rastrollo [31] found no association between high MDS and lower risk of depression in a study of 15,980 adults who were free from depression at baseline and were followed over a median of 10.4 years. Their participants were part of the Seguimiento Universidad de Navarra (SUN) project. Rostami, Parastouei [55] also found no association between MIND diet and symptoms of depression.

##### Stress

Five studies addressed stress or psychological stress. One [57] study found that higher MDS was associated with reduced risk of psychological stress, while four found no association. Salari-Moghaddam, Keshteli [57] studied 3176 Iranian adults who worked in 50 health care centers, excluding those with insufficient or overly abundant caloric intakes (below 800 or above 4200 kcal/d). They found that those in the highest quartile of MDS had lower psychological distress (OR = 0.68; 95% CI: 0.52–0.89) than those in the lowest quartile. However, Barkhordari, Namayandeh [54] did not observe a significant association in their study sample, which enrolled overweight and obese women, nor did Seifollahi, Sardari [56] who studied overweight and obese women (*n* = 282). Rostami, Parastouei [55] also found no statistically significant association in a cohort of male health professionals (*n* = 400).

#### 3.3.8. Multiple Sclerosis

Multiple sclerosis (MS) is an autoimmune central nervous system disease with varying symptoms, such as weakness, numbness, visual disturbance, dizziness, bladder dysfunction, fatigue, and/or bowel dysfunction. Some patients accrue disability over time and may be wheelchair dependent. Thus, this disabling disease may cause a significant impact on their quality of life and the patient’s ability to perform self-care.

Two research groups [27,35] examined the effect of the MIND diet on clinical measures of MS. Katz Sand, Fitzgerald [27] studied the Reserve and Disability in Early Multiple Sclerosis (RADIEMS) cohort, a longitudinal study of risk and protective factors for disability in early MS (<5.0 years). The RADIEMS cohort is based in the U.S. and includes 185 patients, either with relapsing remitting or clinically isolated syndrome, reflecting a typical epidemiology of MS. The sample was primarily female (66.6%) and White (72.2%). The average disease course was 2.2 years, with 21.7% receiving high efficacy disease modifying therapy, and low disability (Expanded Disability Status Scale 1.0). Key MRI variables of the sample population included thalamic volume 21.1 mL (1.7), lesion volume 0.4 mL (1.4), normal appearing white matter 0.0 (0.9) and gray matter volume 804.6 mL (47.8). The MRI of the brain may be used as a diagnostic tool or a measure of the progression of MS. Katz Sand, Fitzgerald examined multiple MRI outcomes. Only participants with high MDS (10–11) experienced a statistically significant correlation with higher thalamic volumes (r = 0.22; 95% CI: 0.07–0.35; *p* = 0.004) even when adjusted for covariates such as disease duration and high-efficacy disease modifying therapy [27]. It should be noted that higher adherence to the MIND diet was an extremely high score of (10–11) compared with other articles that classified a high compliance with the MIND diet as a score ≥ 8.

Noormohammadi, Ghorbani [35] also examined the relationship between the MIND diet and patients with early MS. The authors performed a hospital-based, case–control study in Tehran, Iran, over three years. Newly diagnosed patients with relapsing remitting MS (*n* = 80) were compared with 148 healthy individuals. Most patients were included in the study within the first month of diagnosis of MS and the most common disease modifying therapy was rituximab. To be included, patients must have been diagnosed with MS within the last year, 18–50 years of age, and have an Expanded Disability Status Scale (EDSS) score < 5. Patients were excluded if they experienced a MS relapse within the prior month or lived with another chronic disease (such as chronic kidney disease) that required a specific diet. The study divided adherence to the MIND diet into tertiles: low, medium, or high adherence (actual total score per tertile was not provided). Noormohammadi et al. found that participants with high MDS experienced a 95% reduction in the risk of developing MS compared with participants with low MDS (OR = 0.05, 95% CI = 0.01–0.36, *p* for trend < 0.0001) [35].

Both research groups [27,35] examined the effects of certain foods, such as the proportion of green leafy vegetables or high fat meats, on MS brain metrics and risk of developing MS. The categories of food were vast and difficult to find common ground for comparison. In conclusion, Katz Sand, Fitzgerald [27] found some benefits of the MIND diet associated with only one brain metric, thalamic volume. Noormohammadi, Ghorbani [35] found the MIND diet to have a significantly favorable association with the reduced risk of developing MS.

#### 3.3.9. Parkinson’s Disease

Parkinson’s disease (PD) is the second most common neurodegenerative disorder, and several researchers have explored the impact of the MIND diet on the incidence, progression, and symptoms of PD [62,63,64,65,66]. To date, the findings are equivocal. While earlier studies indicated that greater adherence to the MIND diet was associated with a reduction in the incidence and progression of PD [62], more recent studies have reported no significant association between risk and severity of MIND diet scores and PD [65]. Greater adherence to the MIND diet was associated with later onset of PD (17.4 years; *p* < 0.001) among women [63]. Women reported the highest levels of adherence to the MIND diet. Among men, only adherence to the Greek Mediterranean diet was associated with later onset of PD (8.4 years; *p* = 0.002). Parkinson’s disease is characterized by both motor and nonmotor symptoms. Motor symptoms include tremor, bradykinesia, rigidity, and postural instability. Nonmotor symptoms associated with PD include fatigue, depression, anxiety, apathy, sleep problems, daytime sleepiness, and cognitive impairment. Greater adherence to the MIND diet was associated with decreased total symptom (R^2^ = 0.2207, *p* < 0.001), motor symptom (R^2^ = 0.2344, *p* < 0.001), and nonmotor symptom scores (R^2^ = 0.1849, *p* < 0.001) as measured by the Patient-Reported Outcomes in Parkinson’s disease Scale [64]. However, the evidence to support the positive impact of adherence to the MIND diet on nonmotor symptoms of PD is equivocal given that other studies have found no correlation between MIND diet scores and severity of these symptoms [66]. Most of these studies [63,64,65,66] relied on cross-sectional designs and FFQs. In addition to the limitations inherent in cross-sectional research, FFQs underrepresent the berry food group essential to the MIND diet and have limitations related to recall bias.

### 3.4. Health Outcomes from Single Studies

At the time of this review (early April 2024), each of the health outcomes described below were represented by one article or research group.

#### 3.4.1. Auditory Function

An eight-year longitudinal study of possible associations between diet (as measured by four dietary indices including MIND) and hearing status was conducted by Jin, Tanaka [41] using data from the BLSA. Oxidative stress can promote hearing loss, and diets higher in antioxidants have been linked to preservation of better hearing [76]. The MIND diet recommends foods that optimize antioxidants, so a significant association was anticipated between MDS and hearing. Surprisingly, while Jin et al. found that MDS was linearly correlated with better hearing function, the correlation was weak (*p* < 0.1). While the MIND and Mediterranean diets promote the use of olive oil and fish, the indices do not identify and evaluate the fatty acids as explicitly as the AHEI and the Healthy Eating Index (HEI): healthier diets as measured by both AHEI and HEI were significantly correlated with lower risk of hearing loss.

#### 3.4.2. Glaucoma (Open-Angle)

Glaucoma is a neurodegenerative eye disease resulting in blindness. Plant-based diets have been associated with a lower risk of glaucoma most likely due to higher antioxidant and flavonoid content [28]. Vergroesen, de Crom [28] tested the MIND diet, the Mediterranean diet, and the Dutch dietary guidelines for potential associations with incidence of open angle glaucoma (iOAG) in the Rotterdam Study in the Netherlands. Using a nested case–control design (*n* = 170 cases and *n* = 850 controls), they began enrollment in 1991 and followed up approximately every five years. After 10 years of follow up, participants in the highest quartile of adherence to the MIND Diet (mean adherence in Q1 = 58.8%) had significantly lower risk of iOAG (OR = 0.54; 95% CI = 0.30–0.95) compared with those in the lowest quartile (mean adherence in Q4 = 32.2%). There was not a significant association between the Mediterranean and Dutch dietary guidelines and reduced iOAG.

#### 3.4.3. Irritable Bowel Syndrome

Nouri-Majd, Salari-Moghaddam [53] conducted a cross-sectional study with adults in Iran using data from the Study on the Epidemiology of Psychological, Alimentary Health and Nutrition (SEPAHAN) (*n* = 748) and found no association between the highest levels of adherence to the MIND diet and symptoms of irritable bowel syndrome (IBS). The researchers suspected that participants with IBS (22.2% of the study population) may have avoided some of the foods in the MIND diet, because these foods are high in fermentable oligosaccharides, disaccharides, monosaccharides, and polyols (FODMAPs). FODMAPs can increase symptoms of IBS, and it is common in clinical practice to recommend a low FODMAP diet to people with IBS. Nouri-Majd, Salari-Moghaddam recommend a prospective study to further explore potential associations between the MIND diet and IBS.

#### 3.4.4. Migraine Headaches

Migraine headaches cause significant pain and disability. Askarpour, Yarizadeh [34] studied 266 women from neurology clinics in Tehran, Iran, who suffer from migraines and had a BMI between 18.5 and 30. Using a cross-sectional study design and validated inventories for food intake and migraine symptom, they identified that the odds of having severe headaches were reduced by 36% among those with the highest quartile MDS (OR = 0.64, 95% CI = 0.45–0.91; *p* = 0.01) compared with the lowest quartile. Higher adherence to the MIND diet was also significantly associated with a reduction in the frequency and duration of headaches using linear regression analysis in both crude and adjusted models [34]. While higher adherence to the MIND diet was associated with decreased frequency, duration, and severity of migraine headaches, it was not found to have a significant relationship with disability.

#### 3.4.5. Non-Alcoholic Fatty Liver Disease

Non-alcoholic fatty liver disease (NAFLD) is characterized by excessive accumulation of fat in the liver, often driven by an unhealthy high-calorie diet. In a study of severe NAFLD with patients who were hospitalized or died from NAFLD, diet was measured using five different dietary indices including MDS [61]. As MIND dietary adherence improved, the risk of severe NAFLD significantly decreased using Cox proportional hazard models (*p* < 0.05) [61]. In the first adjusted model, adherence to the MIND diet was significantly associated with lower risk of severe NAFLD in the highest quartile MDS (HR = 0.67, 95% CI = 0.57–0.78, *p* < 0.001). Neither the Mediterranean nor the MIND diets demonstrated statistically significant associations in the third model, which was further adjusted for metabolic phenotypes, while the Mediterranean Diet Adherence Screener, the Recommended Food Score, and the Healthy Diet Indicator demonstrated significant associations.

#### 3.4.6. Quality of Life (Health-Related)

A longitudinal study over 12 years was conducted [23] to identify changes in dietary quality over time as measured by three indices including the MIND diet, the Dietary Inflammatory Index (DII), and the Dietary Guideline Index for Australian Dietary Guidelines (DGI). Analyzing data from the Australian Diabetes, Obesity and Lifestyle study (AusDiab), researchers tested associations between healthier diets and improvement in health-related quality of life (HR-QoL) as defined by the World Health Organization [77]. Ng et al. [23] identified 2844 participants with complete data for baseline, year five and year 12. For all participants, higher MDS was correlated with improved global QoL (β = 0.28, 95% CI = 0.007–0.55). The association between MIND diet and improved HR-QoL was more pronounced among women, who saw improvements in global QoL (β = 0.62, 95% CI = 0.38–0.85), the mental component summary (β = 0.75, 95% CI = 0.29–1.22) and the physical component summary (β = 0.75, 95% CI = 0.29–1.22) [23]. This study demonstrated that increasing adherence to the MIND diet was associated with improved global quality of life. The authors advise that public policies encouraging Australians to implement healthy diets would promote better HR-QoL.

#### 3.4.7. Rheumatoid Arthritis

In a cross-sectional, observational study conducted by Safaei, Kheirouri [67] with 202 total participants (155 women and 47 men), there were 101 participants (mean age 44.79 ± 9.05 years) with rheumatoid arthritis (RA) and 101 healthy subjects (mean age 40.93 ± 7.85). While there was no association between MDS and oxidative stress factors (*p* > 0.05), participants with higher MDS had significantly lower odds of RA than those with lower scores (*p* < 0.001). Additionally, disease activity was lower among those with higher MDS (*p* < 0.05). The findings indicate that following the MIND diet may decrease disease activity and the odds of RA. For patients with RA, a higher adherence to the MIND diet may improve metabolic factors such as lipid profiles and blood glucose levels [67].

#### 3.4.8. Sleep

In a cross-sectional study of health professionals [55], greater adherence to the MIND diet was associated with lower odds of poor sleep (OR = 0.58, 95% CI = 0.34–0.98, *p* = 0.042), daytime sleepiness (OR = 0.58, 95% CI = 0.34–0.98, *p* = 0.044), and insomnia (OR = 0.54, 95% CI = 0.31–0.93, *p* = 0.031), after adjusting for age, BMI, energy intake, smoking status, level of physical activity, marital status, and level of education. However, in addition to the limitations inherent in the cross-sectional design, the study only included male health professionals, limiting generalizability.

#### 3.4.9. Telomere Length

Chan, Leung [68] examined the association of various dietary patterns (Diet Quality Index-International, DASH, MIND diet, Mediterranean diet, Okinawan diet, and Hong Kong diet) with telomere length among Chinese older adults, whose dietary habits and practices differ from those of White populations. They conducted a cross-sectional observational study using multivariate linear regression with available data from 1981 (965 males, 1016 females) of community-dwelling Chinese adults aged 66 years and older living in Hong Kong. Data from an interviewer-administered questionnaire estimated dietary intake and generated dietary patterns. Demographic information, lifestyle factors, and self-reported medical history were also collected. Quantitative real-time PCR was used to measure telomere length. Chan, Leung [68] used a maximum score of 9 instead of 15 for the MIND diet score because they had insufficient information on olive oil as the primary oil source and consumption of fish, beans, poultry, red meat, and fast/fried foods. Participants were primarily female (51.3%), never smokers (65%), never used alcohol (85.3%), had a mean age of 72.4 years, a mean body mass index of 23.6 kg/m^2^, and had primary-school level of education or below (71.8%). The MDS was not associated with telomere length. Chan, Leung [68] speculated that no association between the MIND diet (and the other dietary patterns) and telomere length was observed because the dietary pattern assessment included diluted nutrients or food groups unrelated to telomere length. They suggested that previous studies reported stronger associations between individual nutrients (or food groups) and telomere length, compared with studies examining the association between dietary patterns and telomere length, because the studies on dietary patterns included participants with a broader age range (20–80 years).

## 4. Discussion

In this scoping review, we mapped the extent to which the MIND dietary pattern has been studied relative to health outcomes beyond its original purpose (delaying dementia) and excluding cardiovascular outcomes, which have already been the subject of systematic reviews. The 47 articles included in this review explored 18 different health topics and tested at least 81 different possible associations between high adherence to the MIND dietary pattern and health outcomes (e.g., sleep quality). Sixty-five percent of the tested associations demonstrated a favorable association, representing an improvement in some measure of human health for people who ate at higher levels of adherence to the MIND diet, compared with those people whose MDS were in the lowest tertile, quartile, or other categorical bin.

### 4.1. Associations with the MIND Dietary Pattern

Mostly favorable associations were identified for high adherence to the MIND diet and the following health outcomes: all-cause mortality, BCA, diabetes and metabolic disorders, frailty, functional ability, and PD. The findings from studies included in this review of the MIND dietary pattern are similar to outcomes associated with the Mediterranean diet. This is not surprising, given that the MIND diet is based on the Mediterranean and DASH diets. Systematic reviews of the Mediterranean diet also have demonstrated beneficial outcomes (e.g., reduced incidence or better clinical outcomes) associated with all-cause mortality [78,79], BCA (especially post-menopausal risk reduction) [80], type 2 diabetes [81], frailty [82], physical performance [83], and PD [84].

In this review, where evidence was limited to only one study on a topic, benefits were associated with the MIND diet for the following conditions: glaucoma, migraine headaches, severe NAFLD, HR-QoL, RA, and sleep. Similarly, beneficial associations also have been found between the Mediterranean diet and NAFLD [85], HR-QoL in adults [86] and in children and adolescents [87,88], RA [89], and sleep [90]. The DASH diet also has been found to have beneficial associations with reduction in frequency and severity of migraine headaches [91]. In this review, most of the studies addressing mental health were conducted in Iranian populations, and findings were equivocal for anxiety and depression, whereas there was more evidence for no association between the MIND diet and stress. We recommend that additional studies be conducted in different populations to ascertain whether there is a relationship between the MIND diet and symptoms of anxiety and depression. Previous systematic reviews have identified inverse associations between the Mediterranean diet and incidence and symptoms of anxiety and depression [92].

### 4.2. What Is a High MDS?

Although a meta-analysis was not conducted, it was noted that across the included studies, the average MDS for the highest category of adherence to MIND diet was often modest (e.g., mean of 8.6) when compared with the highest possible score (maximum of 9 to 15) in 46 out of 47 studies that used the original scoring system by Morris, Tangney [2]. This means that people adhering to the MIND dietary pattern approximately 60% of the time, are likely protecting their health more than those who are eating by the MIND dietary pattern less than 50% of the time. It appears that one need not perfectly adhere to the MIND dietary pattern to benefit. By contrast, other associations were only detected at high levels of MDS, such as a mean MDS of 10.68 (±0.81) [27]. The inability to quantify the use of olive oil in some studies may have contributed to lower MDSs and makes comparisons between study outcomes more difficult to achieve. Wine was commonly omitted, and its benefits and risks are debatable [93]. Some studies also identified the unfavorable outcomes associated with the lowest levels of adherence to the MIND dietary pattern; these included diets higher in unhealthy foods (e.g., saturated fats, red meat, and sweets). Finally, several of the studies were conducted in countries (e.g., Iran and China) with common dietary patterns that differ from the MIND diet, such as consumption of wine and the use of olive oil, which affect scoring. In future reviews of the MIND diet, we recommend that data extraction includes noting the average and range of MDS for the bins (e.g., highest tertile or quartile) demonstrating a statistically significant effect (akin to a minimal score necessary to achieve positive results).

### 4.3. Environmental Exposure

In this scoping review, one study identified a deleterious or unfavorable association with the MIND diet: higher cadmium levels in the urine of people with higher adherence to the MIND dietary pattern in an Italian cohort [37]. People are exposed to cadmium primarily through food and cigarette smoke [72,94]. Cadmium occurs naturally, but it is also dispersed by burning fossil fuels, volcanoes, and forest fires [94]. Plants absorb cadmium from the soil and water where they are grown. It would be interesting to know whether environmental exposures of the food supply to cadmium, are relatively higher in that region of Italy where the study was conducted [37]. Many countries monitor foods that may contain cadmium in elevated levels and are consumed by children, such as breakfast cereals, breads, vegetables, and potatoes [72,94,95]. Public health measures should seek ways to reduce exposure to cadmium and other undesirable environmental contaminants (e.g., lead) in healthy foods that are considered beneficial to human health.

### 4.4. MIND Dietary Interventions

Notably missing from the studies included in this scoping review were prospective cohort trials in which the MIND dietary pattern was taught as an intervention. While such studies have been performed to test for effects on cognitive outcomes (i.e., Liu, Morris [96]), the MIND diet has not yet been taught as an educational dietary intervention in the patient populations included in this scoping review. Given the favorable health outcomes associated with the MIND dietary pattern, this is a reasonable next step in scientific and clinical practice, the aim being to learn how to teach and implement the MIND dietary pattern to individuals as well as incorporate healthy eating recommendations into public policy. The MIND dietary pattern guides what and how much to eat from various food groups across the course of a week (e.g., ≥6 servings of leafy greens per week). This simplified guidance may lend itself well to public and clinical translation.

### 4.5. Suggestions for Future Research

We recommend that additional studies in large cohort trials be conducted on the associations identified in this review, particularly with different populations and geographical regions. The MIND diet is a hybrid of two well respected diets, the Mediterranean and DASH diets, and is optimized to increase intake of antioxidants to prevent neurodegeneration and inflammation. There were no prospective cohort trials identified in which the MIND diet was taught as an intervention. In populations that could benefit from healthy dietary intervention, we recommend interventional and translational studies for the purposes of disease prevention and health maintenance. This review included many case–control studies, for which odds ratios (ORs) are often calculated, while studies of different designs measured relative risk or risk ratios (RRs). Odds ratios usually report larger values compared to risk ratios (RRs) [97]. For example, in this review, the average RR was around 25% while the average OR was 50%. Given the type of analysis and the study design, it may be that case–control studies overestimate the impact of the MIND diet. Thus, more prospective studies are needed to confirm the impact of the MIND dietary pattern on the health outcomes included in this review.

### 4.6. Limitations of the Review

This study has some limitations. As with other reviews, we concluded our search of the literature to conduct the review, such that articles published since April 2024 were not included. Most of the studies included relied upon FFQs to calculate MDS. While some of the various FFQs used were culturally appropriate to specific populations, FFQs have fallen under strong criticism for their potential inaccuracies and biases, namely recall, social desirability, response, and misclassification biases that can significantly impact the conclusions drawn [98]. The decision was made not to replicate what had already been addressed by previous systematic reviews, so articles addressing cognition, dementia, and cardiovascular health were omitted, unless these studies addressed other health outcomes such as all-cause mortality. It is possible that we missed several important studies by excluding these topics. At the time of writing, we learned that a cardiovascular review had been retracted by the publisher due to errors in methods and analyses. From this experience, we learned that when only one systematic review has been published on a topic, there could be a benefit to duplicating the work, even though a scoping review is considered less rigorous. Finally, we followed the methodology of a scoping review as defined by PRISMA-ScR [20], which means that no quality analyses or meta-analyses were conducted. At the time of this project, there were insufficient studies published on any single topic to conduct a systematic review. We anticipate opportunities for additional systematic reviews of the MIND diet in the future.

### 4.7. Conclusions

This scoping review identified new lines of inquiry concerning MIND dietary adherence and human health. The findings of the present study provide preliminary evidence that the MIND diet may be associated with lowering the odds of developing several different chronic diseases and/or lessening the symptoms associated with some diseases and conditions (all-cause mortality, BCA, diabetes and metabolism, functional ability, and PD). High adherence to the MIND dietary pattern was associated with only one unfavorable outcome, cadmium exposure, which depended upon environmental conditions and farming practices where plants were grown. This risk is not unique to the MIND diet. It is essential worldwide to ensure a safe supply of healthy foods that are beneficial to human health.

## Figures and Tables

**Table 1:** The MIND dietary pattern, adapted from descriptions by Morris [9].

**10 Healthy Foods**	**Servings/Frequency**	**Examples**
Leafy green vegetables	6+/week	Kale, spinach, romaine lettuce
Other vegetables	1+/day	Broccoli, green beans, squash
Whole grains	3+/day	Oats, farro, wheat, brown rice
Berries	2+/week	Blueberries, strawberries
Nuts	5+/week	Walnuts, almonds, peanuts
Seafood	1+/week	Fish, shrimp, scallops (fresh or saltwater fish)
Poultry	2+/week	Chicken, turkey
Beans & legumes	4+/week	Black beans, lentils
Olive oil as primary oil		
Wine *	1/day for women 1–2/day for men	5 ounces of wine, 12 ounces of beer (5% alcohol), or 1.5 ounces of liquor
**5 Unhealthy Foods to Limit**	**Servings/Frequency**	**Examples**
Sweets & pastries	<5/week	Cake, candy, ice cream, pie
Red meats & red-meat products	<4/week	Beef, pork, bacon, corn beef hash
Fried/fast foods	<1/week	Fries, burgers, chicken
Whole-fat cheese	<1/week (1 to 2 ounces per week)	Cheddar, Colby, Swiss, American
Butter or trans-fat margarine	<1 pat/day	Butter, margarine

* The original MIND diet includes wine; however, people who do not consume alcohol are not encouraged to begin.

**Table 2:** Keywords used.

“MIND diet”
“MIND dietary pattern”
“Mediterranean DASH Intervention for Neurodegenerative Delay”
“Mediterranean Dietary Approaches to Stop Hypertension Intervention for Neurodegenerative Delay”
Filters applied: Publication Date from 2015 to 2024

**Table 3:** Statistically significant associations with the MIND diet.

Number of Studies	Health Outcome	Favorable n (%)	Unfavorable n (%)	No Association n (%)	Study Reference(s)
6	All-Cause mortality:	4 (67%)	0	2 (33%) *	Song [26]; Song [38]; Thomas [25]; Zupo [39]; Corley [30]; Chan [40]
	All-cause mortality in a diabetic population	1	0	0	Song [26]
	All-cause mortality (non-diabetic population)	1	0	0	Song [26]
	All-cause mortality in a hypertensive population	1	0	0	Song [38]
1	Auditory function	0	0	1 (100%) *	Jin [41]
5	Cancer (Risk of):				
	Breast cancer	2 (67%)	0	1 (33%)	Mokhtari [36]; Aghamohammadi [42]; Sheikhhossein [43]
	Glioma	2 (100%)	0	0	Zhang [33]; Soltani [44]
2	Diabetes and metabolism:				
	Metabolic health status	1 (100%)	0	0	Tirani [45]
	Hypertension	1 (100%)	0	0	Tirani [45]
	Hypertriglyceridemia	1 (100%)	0	0	Tirani [45]
	Serum brain-derived neurotrophic concentrations	0	0	1 (100%)	Tirani [45]
	Incident diabetes	1 (100%)	0	0	Tison [46]
3	Dietary exposures:				
	Sulfur content in food	1 (100%)	0	0	Passafiume [47]
	Selenium exposure	1 (100%)	0	0	Urbano [48]
	Cadmium exposure	0	1 (100%)	0	Urbano [37]
2	Frailty (risk of)	2 (100%)	0	0	Yao [32]; Tanaka [24]
6	Functional ability:				
	ADL disability	1 (100%)	0	0	Agarwal [49]
	Instrumental ADL disability	1 (100%)	0	0	Agarwal [49]
	Mobility disability	1 (100%)	0	0	Agarwal [49]
	Grip strength	2 (100%)	0	0	Pasdar [50]; Talegawkar [51]
	Physical function	1 (100%)	0	0	Talegawkar [51]
	Psychological function	1 (100%)	0	0	Yeung [52]
1	Glaucoma (open-angle)	1 (100%)	0	0	Vergroesen [28]
1	Irritable bowel syndrome	0	0	1 (100%)	Nouri-Majd [53]
10	Mental health:				
	Anxiety	2 (40%)	0	3 (60%)	Barkhordari [54]; Rostami [55]; Seifollahi [56]; Salari-Moghaddam [57]; Torabynasab [58]
	Depression	3 (50%)	0	3 (50%) *	Barkhordari [54]; Cherian [29]; Fresan [31]; Seifollahi [56] *; Salari-Moghaddam [57]; Rostami [55]
	Impulsivity	0	0	1 (100%)	Gomez-Martinez [22]
	Mood	0	0	1 (100%)	Ma [59]
	Somatization	1 (100%)	0	0	Haghighatdoost [60]
	Stress	1 (20%)	0	4 (80%)	Barkhordari [54]; Rostami [55]; Salari-Moghaddam [57]; Seifollahi [56]
1	Migraine headaches:				
	Severity	1 (100%)	0	0	Askarpour [34]
	Frequency	1 (100%)	0	0	Askarpour [34]
	Duration	1 (100%)	0	0	Askarpour [34]
	Disability	0	0	1 (100%)	Askarpour [34]
2	Multiple sclerosis:				
	Reduced odds of MS	1 (100%)	0	0	Noormohammadi [35]
	Higher thalamic volume	1 (100%)	0	0	Noormohammadi [35]
	Lesion volume	0	0	1 (100%)	Katz Sand [27]
	Gray matter volume	0	0	1 (100%)	Katz Sand [27]
	Normal appearing white matter	0	0	1 (100%)	Katz Sand [27]
1	Non-alcoholic fatty liver disease	1(100%)	0	0	Petermann-Rocha [61]
5	Parkinson’s disease:				
	Incidence	1 (100%)	0	0	Agarwal [62]
	Later onset	1 (100%)	0	0	Metcalfe-Roach [63]
	Motor symptoms	1 (100%)	0	0	Fox [64]
	Nonmotor symptoms	1 (100%)	0	0	Fox [64]
	Progression	1 (100%)	0	0	Agarwal [62]
	Risk	0	0	1 (100%)	Keramati [65]
	Severity	0	0	2 (100%)	Keramati [65]; Lawrie [66]
	Total symptoms	1 (100%)	0	0	Fox [64]
1	Quality of Life (health-related)	1 (100%)	0	0	Ng [23]
1	Rheumatoid arthritis:				
	Oxidative stress indicators	0	0	1 (100%)	Safaei [67]
	Metabolic factors	1 (100%)	0	0	Safaei [67]
	Disease activity	1 (100%)	0	0	Safaei [67]
	Odds of disease	1 (100%)	0	0	Safaei [67]
1	Sleep:				
	Sleep quality	1 (100%)	0	0	Rostami [55]
	Insomnia	1 (100%)	0	0	Rostami [55]
	Sleepiness (daytime)	1 (100%)	0	0	Rostami [55]
1	Telomere length	0	0	1 (100%)	Chan [68]

*n* = number. Percentages were calculated based on the number of studies reporting on the same health outcome. * A weak but favorable association was found in one or more studies.

## Supplementary Materials

The following supporting information can be downloaded at: https://www.mdpi.com/article/10.3390/nu17162687/s1, Table S1: Characteristics of included articles.
